# Inhibition of ZDHHC16 promoted osteogenic differentiation and reduced ferroptosis of dental pulp stem cells by CREB

**DOI:** 10.1186/s12903-024-04107-x

**Published:** 2024-03-26

**Authors:** Wei Liu, Wenwei Yu, Lili Zhou, Danhua Ling, Yangbo Xu, Fuming He

**Affiliations:** 1 Department of Oral Medicine, the Second Affiliated Hospital of Zhejiang University School of Medicine, 88 Jiefang Road, Hangzhou, Zhejiang 310009 China; 2 Department of Oral Prosthodontics, Stomatology Hospital, School of Stomatology, Key Laboratory of Oral Biomedical Research of Zhejiang Province, Zhejiang University School of Medicine, Zhejiang Provincial Clinical Research Center for Oral Disease, 166 Qiu’tao Road (N), Hangzhou, Zhejiang 310000 China; 3 Department of General Dentistry, the Second Affiliated Hospital of Zhejiang University School of Medicine, 1511 Jianghong Road, Hangzhou, Hangzhou, Zhejiang 310052 China

**Keywords:** ZDHHC16, CREB, Dental pulp stem cells, Ferroptosis, Periodontitis

## Abstract

**Background:**

The repair of bone defects caused by periodontal diseases is a difficult challenge in clinical treatment. Dental pulp stem cells (DPSCs) are widely studied for alveolar bone repair. The current investigation aimed to examine the specific mechanisms underlying the role of Zinc finger DHHC-type palmitoyl transferases 16 (ZDHHC16) in the process of osteogenic differentiation (OD) of DPSCs.

**Methods:**

The lentiviral vectors ZDHHC16 or si-ZDHHC16 were introduced in the DPSCs and then the cells were induced by an odontogenic medium for 21 days. Subsequently, Quantitate Polymerase Chain Reaction (PCR), immunofluorescent staining, proliferation assay, ethynyl deoxyuridine (EdU) staining, and western blot analysis were used to investigate the specific details of ZDHHC16 contribution in OD of DPSCs.

**Results:**

Our findings indicate that ZDHHC16 exhibited a suppressive effect on cellular proliferation and oxidative phosphorylation, while concurrently inducing ferroptosis in DPSCs. Moreover, the inhibition of ZDHHC16 promoted cell development and OD and reduced ferroptosis of DPSCs. The expression of p-CREB was suppressed by ZDHHC16, and immunoprecipitation (IP) analysis revealed that ZDHHC16 protein exhibited interconnection with cAMP-response element binding protein (CREB) of DPSCs. The CREB suppression reduced the impacts of ZDHHC16 on OD and ferroptosis of DPSCs. The activation of CREB also reduced the influences of si-ZDHHC16 on OD and ferroptosis of DPSCs.

**Conclusions:**

These findings provide evidences to support a negative association between ZDHHC16 and OD of DPSCs, which might be mediated by ferroptosis of DPSCs via CREB.

**Supplementary Information:**

The online version contains supplementary material available at 10.1186/s12903-024-04107-x.

## Introduction

Periodontitis is a chronic and progressive condition that is primarily triggered by dental plaque and results in damage to the periodontal support tissue [1]. This disease can destroy the supporting bone tissue structure of the teeth and eventually lead to the loosening and falling of the teeth [2]. The existing periodontal treatment methods cannot achieve complete restoration of periodontal tissue [3]. The so-called “endogenous” is only for the source of cells and does not exclude other therapeutic interventions. There is still a great deal of work to do in order to regenerate tooth and periodontal damage [4].

Tissue engineering is an interdisciplinary area of study that involves the integration of cells or proteins with biomaterials to create novel tissue structures. Bone tissue regeneration has always been the direction of scholars’ efforts. Adult stem cells refer to a population of undifferentiated cells present in tissues or organs of adult organs [5]. These cells possess the ability to undergo self-renewal and differentiate into diverse cell types [6]. Stem cells isolated from various tissues have different biological characteristics and different osteogenic differentiation (OD) abilities [7]. Among them, the stem cells derived from bone marrow are the most studied, and the dental pulp stem cells (DPSCs) are also widely studied because of their excellent OD potential and accessibility [8, 9]. Several previous studies have shown that under the control of growth factors, transcription factors, and receptor molecules, DPSCs can differentiate into osteoblasts, adipocytes, odontoblasts, and nerve cells [10, 11]. Also, DPSC has the potential to regenerate periodontal tissue [12]. Additionally, DPSCs offer the following advantages: 1). There are abundant sources of DPSCs because children’s teeth that fall out naturally and wisdom teeth that need to be extracted by adults are rich in DPSCs. 2). Mesenchymal stem cells and DPSCs share many characteristics. It is possible to use DPSCs without strict matching due to their low immunogenicity and minimal impact on immune function [13]. Hence, due to their heterogeneity, DPSCs represent a promising option for regenerative medicine and clinical therapy. However, the precise mechanisms through which DPSCs regulate the intricate equilibrium among proliferation, differentiation, and self-renewal have yet to be comprehensively elucidated. The mechanism by which DPSCs promote periodontal tissue regeneration and periodontitis healing is still unclear.

Ferroptosis is a novel mode of cell death, characterized primarily by Fe^2+^ level rise, Reactive oxygen species (ROS) accumulation, a decrease in glutathione (GSH) synthesis, and the increase in lipid peroxidation products, leading to cell membrane damage and even cell death. Solute carrier family 7 member 11 (SLC7A11) is a component of the cystine/glutamic acid reverse transporter, which participates in GSH synthesis. Inhibition of SLC7A11 expression can reduce GSH activity [14–17]. Previous research studies have revealed that p53 can promote ferroptosis by inhibiting the expression of SLC7A11 [18, 19]. Ferroptosis has been implicated in various medical conditions, and recent study has indicated a potential connection between ferroptosis and the progression of microbial infectious diseases [20]. But the mechanism of ferroptosis in periodontal disease has not yet been elucidated.

cAMP-response element binding protein (CREB) is further activated and promotes the release of neurotransmitters in synaptic gaps by regulating the formation and development of neural dendrites, intervening in the proliferation and differentiation of neural cells. CREB is involved in regulating various intracellular signal transduction pathways in neurons and mediates neuroprotective effects [21]. The CREB pathway is an important signal pathway downstream of the G protein-coupled receptor, which has a vital role in inducing the osteoblastic differentiation of stem cells, such as DPSCs [22].

Stem cells are differentiated by post-translational modifications [23]. S- palmitoylation, a significant post-translational modification, plays a crucial role in regulating protein targeting, trafficking, and stability. This modification is facilitated by a conserved sequence motif, Asp-His-His-Cys (DHHC), which was found in 23 mammalian palmitoyl acyltransferases responsible for catalyzing S-palmitoylation [24]. . Recent research has underscored the extensive participation of ZDHHCs in the progression of various diseases. Enhancing glioma malignancy, ZDHHC15 emerged as a potential novel prognostic biomarker for glioma patients [25]. Palmitoylation played a crucial role in enhancing osteoclast differentiation and activity, presenting itself as a plausible therapeutic target for addressing osteoporosis and other diseases associated with osteoclast dysfunction [26]. But there is no data describing how DHHCs affect DPSCs osteogenic differentiation, despite the fact that numerous proteins are modified by palmitoylation. As a member of the ZDHHC family, ZDHHC16 has a significant role in palmitoylation. It can promote the addition of palmitate to various protein substrates, thus regulating the function of related proteins [27]. In ovarian cancer, ZDHHC16 was the dominant enzyme for palmitoylation by claudin 3 (CLDN3) [28]. Through palmitoylation, ZDHHC16 stabilizes the CLDN3 protein and promotes the incidence and growth of ovarian cancer [29, 30]. In glioblastoma, palmitoylation of SET domain-containing 2 (SETD2), mediated by ZDHHC16, protected SETD2 from degradation. This process facilitated its role in mediating DNA damage response and suppressing cancer initiation [31]. A recent study has shown that the ZDHHC16 gene inhibited OD of bone marrow mesenchymal stem cells (BMSCs) [27], so this may also be a potential regulatory target for OD of DPSCs.

Consequently, the current investigation examined the particular contribution details of ZDHHC16 to OD of DPSCs. This was the first attempt to discuss the connection between ZDHHC16 and OD of DPSCs, with an attempt to analyze its specific mechanisms. We hypothesized that inhibition of ZDHHC16 can promote OD and reduce ferroptosis of DPSCs through the CREB pathway. This hypothesized effect could have a positive impact on regeneration of alveolar bone or treatment of bone loss of periodontitis.

## Materials & methods

### Cell culture and RNA interference

DPSCs(PC-026 h; Saios, Wuhan, Hubei, China)were cultured as described previously [32]. DPSCs were cultivated in a 6-cm dish employing Dulbecco’s modified Eagle’s medium, which was treated with 10% fetal bovine serum and 1% penicillin/streptomycin. The cells were maintained at 37 °C and a CO_2_ concentration of 5%. Lentiviral vectors containing ZDHHC16 (sc-413,614; Santa Cruz Biotechnology, CA, USA) or si-ZDHHC16 (sc-90,591; Santa Cruz Biotechnology, CA, USA) were introduced into DPSCs. After 48 h of transfection, DPSCs were induced by an odontogenic medium. Following a 21-day period of induction via an odontogenic medium, cells were fixed and subsequently stained with alkaline phosphatase (ALP).

### Quantitate PCR

TRIZOL reagent (Life Technologies corporation, Gaithersburg, MD, USA) was utilized to extract total RNA from cell samples. The qRT-PCR assay was conducted using Light Cycler 480 SYBR Mix (Roche, Basel, Switzerland) on the Light Cycler 480 real-time PCR system. The ^2−ΔΔct^ method was utilized to normalize the mRNA expression levels to the GAPDH expression. The primers used were as follows: ZDHHC16 F: 5′- ACTGGCTGGTAGACAACGTG − 3′; ZDHHC16 R: 5′- TGGCGATATCATTCCTGCCC − 3′; Runt-related transcription factor 2 (Runx2) F: 5′- GACTGTGGTTACCGTCATGGC-3; Runx2 R: 5′- ACTTGGTTTTTCATAACAGCGGA − 3′; Osteocalcin (OCN) F: 5′- GACAAGTCCCACACAGCAACT − 3′; OCN R: 5′- GGACATGAAGGCTTTGTCAGA − 3′; Collagen type I alpha 1 chain (Col1a1) F: 5′- GCTCCTCTTAGGGGCCACT − 3′; Col1a1 R: 5′- ATTGGGGACCCTTAGGCCAT − 3′; Osterix (OSX) F: 5′-TTCTGCGGCAAGAGGTTCACTC-3′; OSX R: 5′-GTGTTTGCTCAGGTGGTCGCTT-3′.

### Immunofluorescent staining

The cells were subjected to a 24-h incubation period in an environment of 5% O2, 5% CO2, and 90% N2. Subsequently, the cells were treated with 4% paraformaldehyde for 15 min, followed by incubation with 0.15% Triton X 100 for 15 min at 37 °C. Consequently, the cells were subjected to incubation with ZDHHC16 (1:500, Abcam, Cambridge, MA, USA) and p-CREB (1:500, Cell Signaling Technology, Danvers, MA, USA) at 4 ˚C for an overnight duration subsequent to blocking with 5% BSA for 60 min. The cells were then subjected to incubation with either goat anti-rabbit IgG-cFL 488(sc-362,262; Santa Cruz Biotechnology, CA, USA) or anti-rabbit IgG-cFL 555(sc-516,249; Santa Cruz Biotechnology, CA, USA) at a dilution of 1:100 for 2 h at room temperature. Subsequently, the cells were stained with DAPI for 15 min, followed by a rinse with PBS for 15 min. Ultimately, the cellular imagery was acquired utilizing a fluorescent microscope (Axioplan 2; Carl Zeiss, Oberkochen, Germany).

### Proliferation assay, EDU staining and cell migration assay

Following a 48-h transfection period, a quantity of roughly 2 × 10^3^ cells/well was seeded into a 96-well plate for the purposes of conducting cell counting kit-8 (CCK-8) analysis. Consequently, cellular proliferation was assessed by means of the CellTiter-GloR Luminescent Cell Viability Assay (Promega, Madison, WI, USA.), following the manufacturer’s directions, after cultivating for the specified time intervals (0, 1, 2, 3, and 4 days).

In the ethynyl deoxyuridine (EdU) incorporation assay, 10 mM EdU was introduced into each well, followed by cell fixation with 4% formaldehyde for 30 min. Following the washing procedure, EdU was identified utilizing the Click-iTR EdU Kit (Invitrogen, Carlsbad, CA, USA) and subsequently visualized through a fluorescent microscope (BX51; Olympus, Tokyo, Japan).

The chamber system was utilized for quantifying the overall migration across the entire cross-section. These cells were introduced into 24-well plates, and 30 µl of 3 × 10^4^ cells in serum-free Dulbecco’s Modified Eagle Medium (DMEM) were applied to the upper chamber. The lower chamber of each well received DMEM supplemented with 10% fetal bovine serum (FBS), and the cells were then incubated for 24 h. The cells on the lower side of the membrane were methanol-fixed and subjected to staining with 0.1% crystal violet. Ultimately, the cell migration through the chamber was captured using a microscope (Axioscope; Carl Zeiss, Oberkochen, Germany). Enumerate the migrated cells in a minimum of three distinct regions.

### Western blot

The tissue or cellular specimens were subjected to lysis employing ice-cold RIPA buffer supplemented with complete protease and phosphatase suppressors. Consequently, the BCA protein assay kit (Abcam, Cambridge, MA, USA) was utilized to quantify the protein concentrations. The process of splitting total proteins was executed through the utilization of SDS-PAGE, followed by the transfer of these proteins onto polyvinylidene difluoride (PVDF) membranes. Following the blocking of membranes with 5% BSA in TBS, primary antibodies including ZDHHC16 (1:1000, Abcam, Cambridge, MA, USA), CREB (1:1000, Cell Signaling Technology, Danvers, MA, USA ), p-CREB (1:1000, Abcam, Cambridge, MA, USA), glutathione peroxidase 4 (GPX4) (1:1000, Abcam, Cambridge, MA, USA) and β-Actin (1:5000, Santa Cruz Biotechnology, CA, USA ) were incubated. Subsequently, horseradish peroxidase-conjugated secondary antibodies (Santa Cruz Biotechnology, CA, USA ) were incubated with the membranes. The ECL system was utilized to determine the signals, which were subsequently exposed by the ChemiDoc XRS system with Image Lab programs (Bio-Rad, Hercules, CA, USA). The samples derived from the same experiment and that gels were processed in parallel.

### Statistical analyses

The study employed the Kaplan-Meier method and log-rank test to conduct survival analysis on the data. The statistical analysis was conducted using GraphPad Prism 6 (GraphPad, CA, USA). A statistical significance level of *P* < 0.05 was considered appropriate. Statistical analysis involved the utilization of Student’s t-test or one-way analysis of variance (ANOVA) to compare data across groups, followed by Tukey’s post hoc test.

## Results

### The inhibition of ZDHHC16 promoted cell growth of DPSCs

**Fig. 1:**
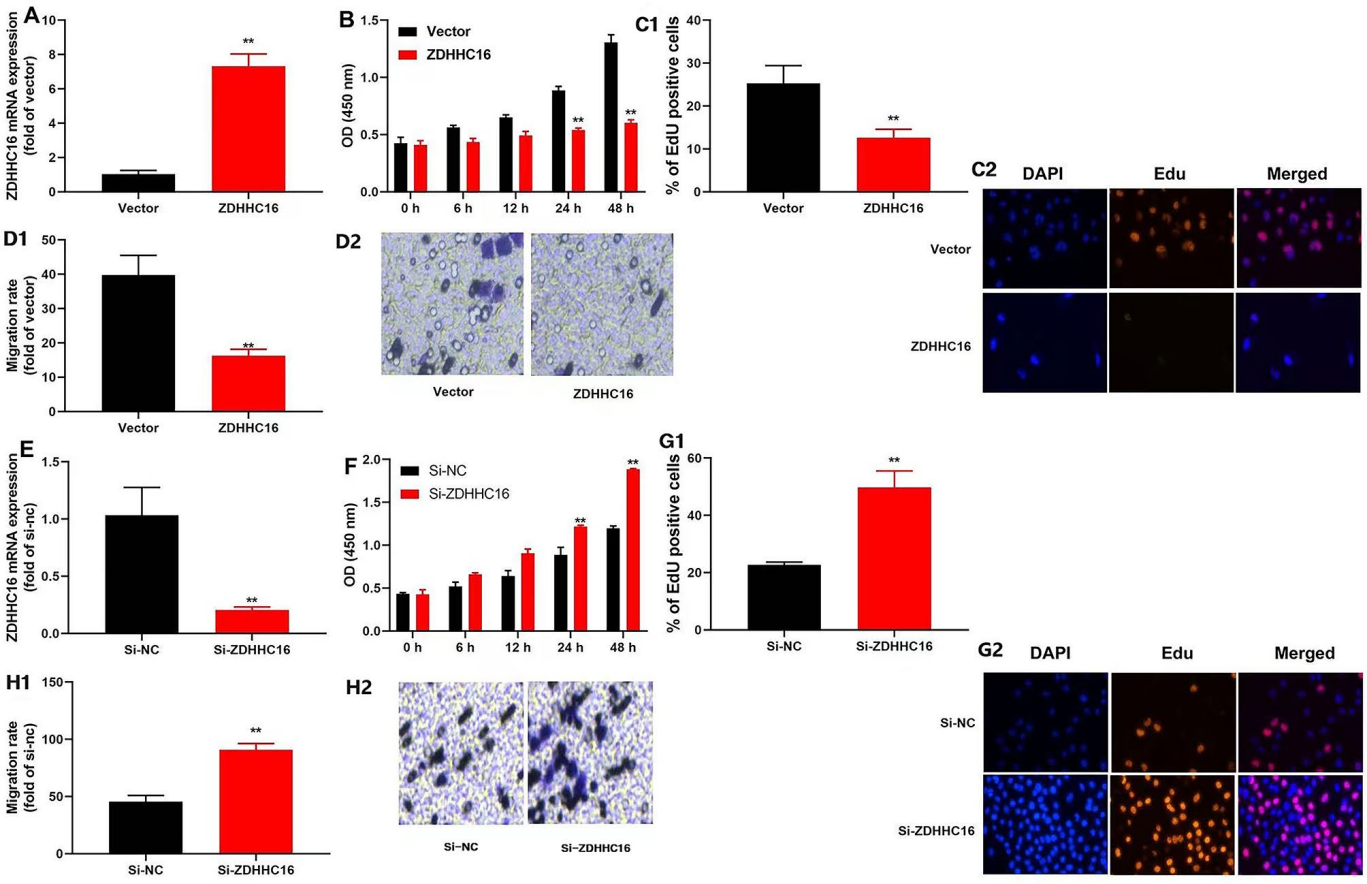
The inhibition of ZDHHC16 promoted cell growth of DPSCs ZDHHC16 plasmid increased ZDHHC16 mRNA expression, and si-ZDHHC16 plasmid reduced ZDHHC16 mRNA expression in DPSCs (**A** and **E**); ZDHHC16 up-regulation decreased cell development, and ZDHHC16 down-regulation increased cell development (**B** and **F**); ZDHHC16 up-regulation decreased the number of EdU cells, and ZDHHC16 down-regulation promoted the quantity of EdU cells (×20, C1, C2 and G1, G2); ZDHHC16 up-regulation inhibited the migration rate of DPSCs, and ZDHHC16 down-regulation promoted the DPSCs migration rate(×50, D1, D2 and H1, H2). **p < 0.01 compared with negative or si-nc.

First, we confirmed the effect of ZDHHC16 on the cell growth of DPSCs. Our data revealed that ZDHHC16 plasmid increased ZDHHC16 mRNA expression, and si-ZDHHC16 plasmid reduced ZDHHC16 mRNA expression in DPSCs **(**Fig. 1A **and E)**. Furthermore, we found that up-regulation of ZDHHC16 decreased cell development and the number of EdU cells and inhibited the migration rate of DPSCs **(**Fig. 1B–D**)**. Meanwhile, ZDHHC16 down-regulation promoted the DPSCs migration rate and increased cell growth and the quantity of EdU cells **(**Fig. 1F–H**)**. Overall, the findings indicated that ZDHHC16 functioned as a reparative factor in the cellular proliferation of DPSCs.

### ZDHHC16 suppression promoted OD of DPSCs

**Fig. 2:**
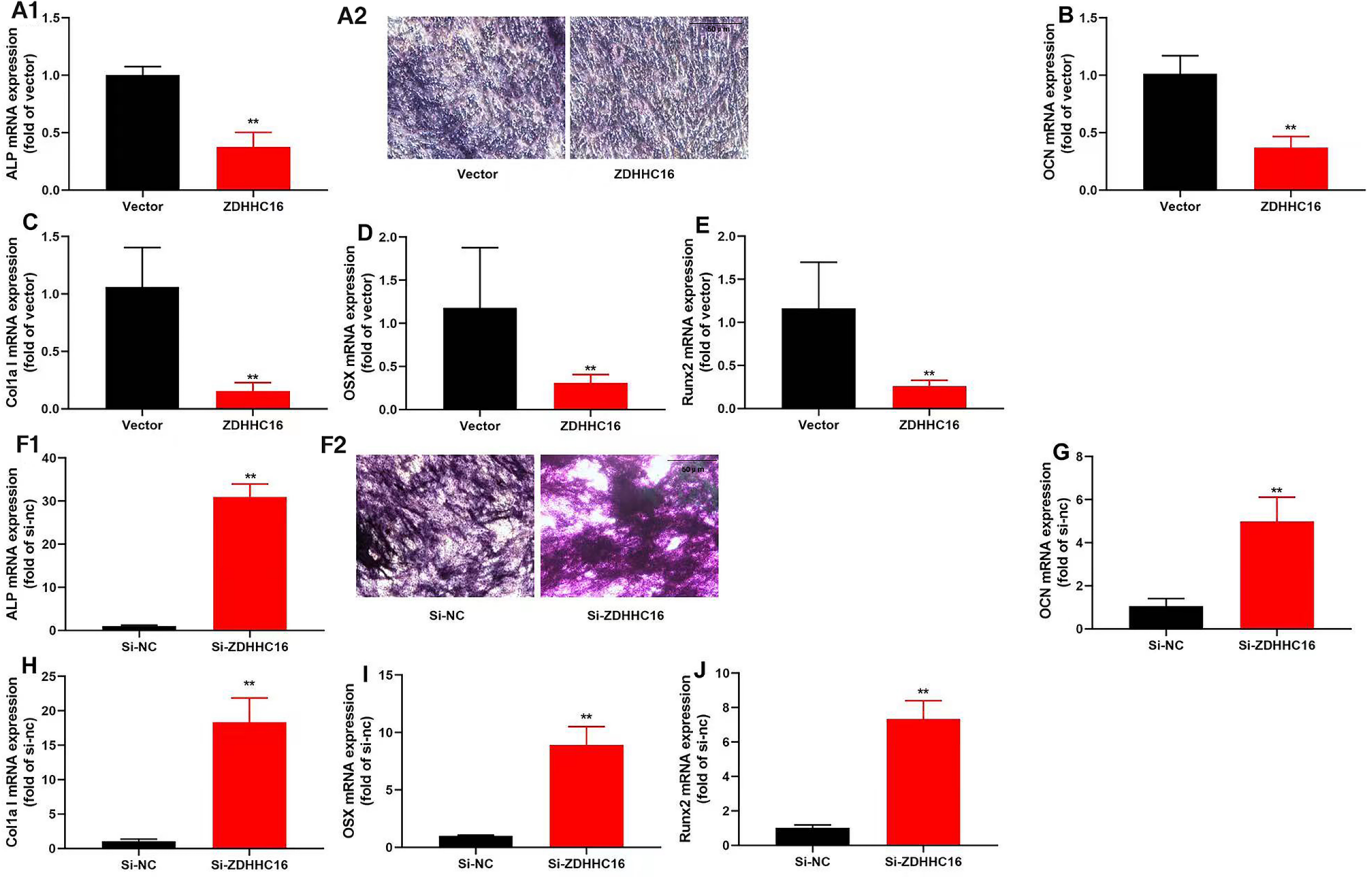
The inhibition of ZDHHC16 promoted Osteogenic differentiation of DPSCs ALP mRNA expression and staining was decreased (×50, A1, A2), and OCN (B), Col1a1 (C), OSX (D), Runx2(E) mRNA expression in DPSCs were inhibited by ZDHHC16 up-regulation; ALP mRNA expression and staining was increased (×50, F1, F2), and OCN (G), Col1a1 (H), OSX (I), Runx2 (J) mRNA expression in DPSCs were promoted by ZDHHC16 down-regulation. **p < 0.01 compared with negative or si-nc.

Herein, we determined the impacts of ZDHHC16 on the OD of DPSCs. Our results demonstrated that upregulation of ZDHHC16 inhibited the expression levels of OCN, ALP, OSX, Col1a1, and Runx2 mRNA and decreased the levels of ALP activity in DPSCs **(**Fig. 2A–E**).** Therefore, ZDHHC16 down-regulation promoted Col1a1, ALP, OSX, OCN, and Runx2 mRNA expression levels and increased ALP activity levels in DPSCs **(**Fig. 2F–J**)**. Furthermore, our findings revealed that The ZDHHC16 inhibition promoted the OD of DPSCs.

### ZDHHC16 inhibition decreased ferroptosis of DPSCs

**Fig. 3:**
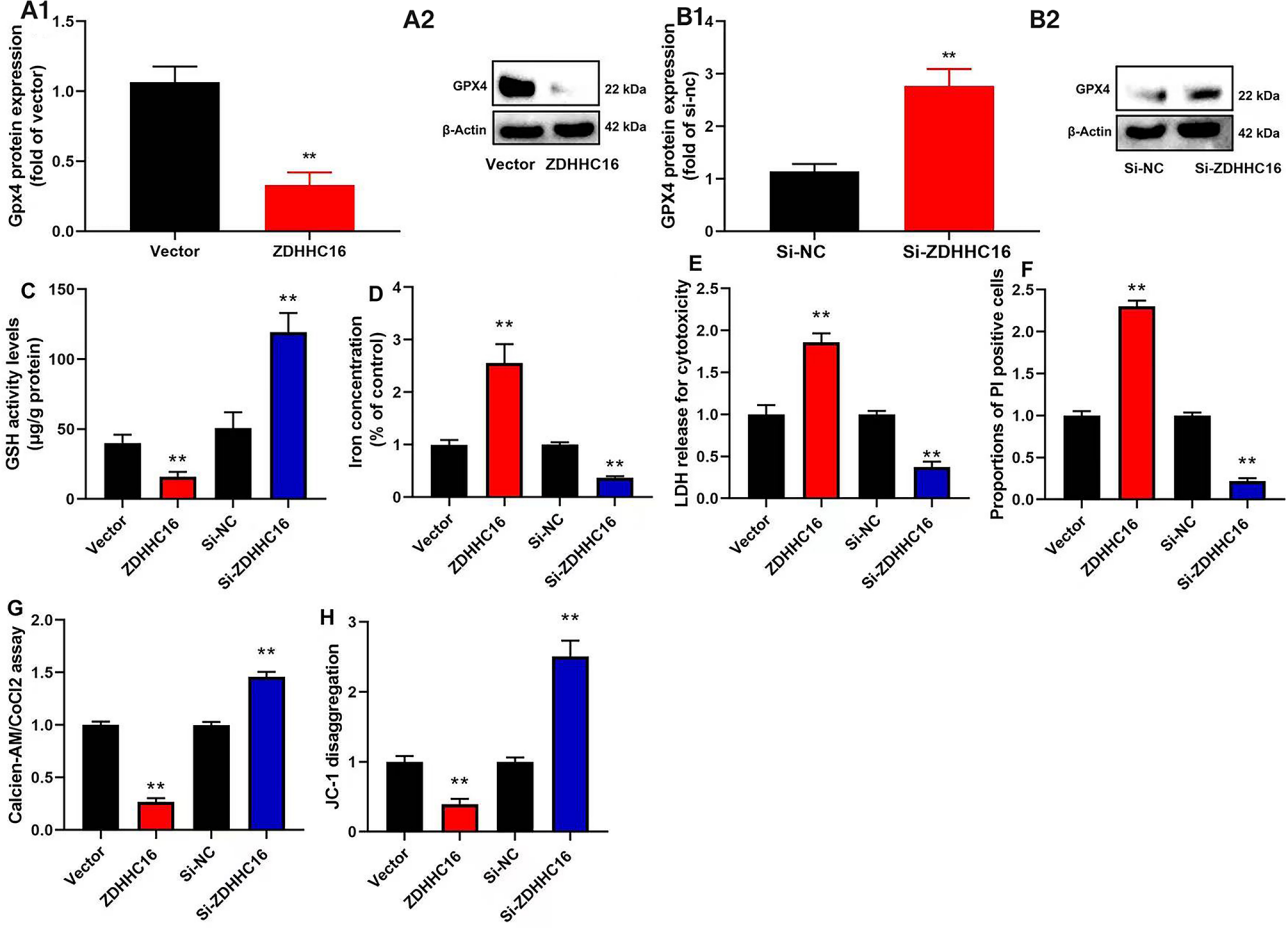
The inhibition of ZDHHC16 reduced Ferroptosis of DPSCs GPX4 protein expression (A1, A2), GSH activity (**C**), MPT (calcein AM/CoCl2 assay, **G**), JC-1 disaggregation (**H**) were inhibited by up-regulation of ZDHHC16, and iron concentration (**D**), LDH activity level (**E**), PI positive cells (**F**) were increased by up-regulation of ZDHHC16; GPX4 protein expression (B1, B2), GSH activity (**C**), MPT (calcein AM/CoCl2 assay, G), JC-1 disaggregation (**H**) were increased by down-regulation of ZDHHC16, and iron concentration (**D**), LDH activity level (**E**), PI positive cells (**F**) were decreased by down-regulation of ZDHHC16. **p < 0.01 compared with negative or si-nc.

Additionally, we examined how the mechanism of ZDHHC16 affected the ferroptosis of DPSCs. Up-regulation of ZDHHC16 inhibited GSH activity levels and GPX4 protein expression in DPSC **(**Fig. 3A **and C)**. We revealed that down-regulation of ZDHHC16 resulted in induction of the expression of the GPX4 protein and increased levels of GSH activity in DPSC **(**Fig. 3B–C**)**. Moreover, the upregulation of ZDHHC16 increased iron concentration and lactate dehydrogenase (LDH) level, increased PI-positive cells, and reduced JC-1 disaggregation and MPT (calcein AM/CoCl2 assay) of DPSCs **(**Fig. 3D–H**)**. Additionally, we reported that down-regulation of ZDHHC16 resulted in a reduction in iron and LDH activity levels, a decrease in the number of PI-positive cells, an increase in JC-1 disaggregation, and an increase in MPT (calcein AM/CoCl2 assay) of DPSCs **(**Fig. 3D–H**)**. Ultimately, we confirmed that the inhibition of ZDHHC16 reduced the ferroptosis of DPSCs.

### ZDHHC16 suppressed p-CREB expression

**Fig. 4:**
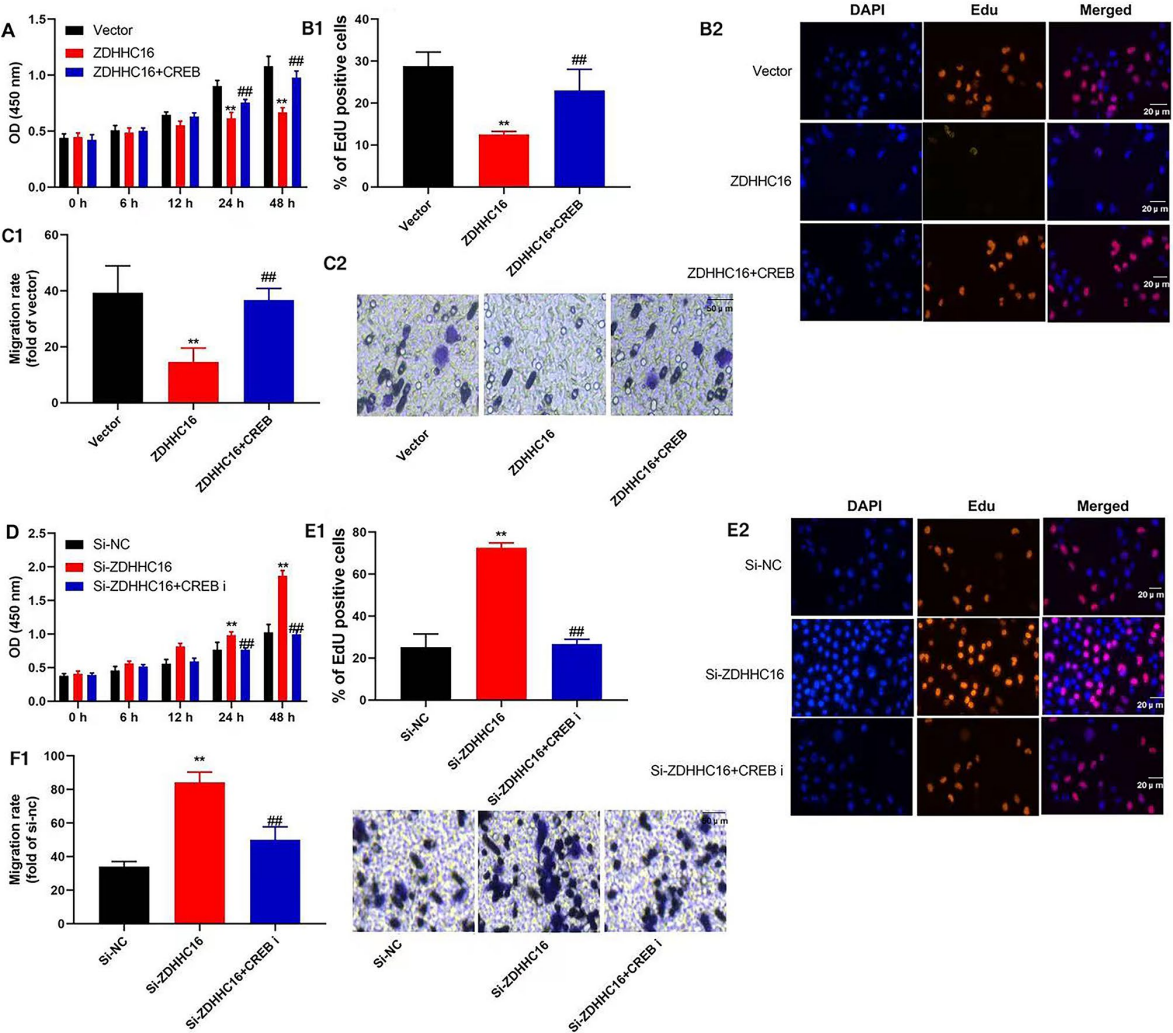
ZDHHC16 suppressed p-CREB expression ZDHHC16 protein expression was promoted, and p-CREB protein was inhibited by ZDHHC16 plasmid of the DPSCs (A1, A2, A3); ZDHHC16 protein expression was inhibited, and p-CREB protein was promoted by Si-ZDHHC16 plasmid of the DPSCs (B1, B2, B3); ZDHHC16 expression was induced, and p-CREB expression was suppressed by ZDHHC16 plasmid of DPSCs (×200, Immunofluorescence, C), ZDHHC16 protein interlinked CREB protein (D), CREB agonist induced p-CREB and GPX4 protein expressions of DPSCs by ZDHHC16 up-regulation (E1, E2,E3); CREB inhibitor suppressed p-CREB and GPX4 proteins of DPSCs by down-regulation of ZDHHC16 (F1, F2, F3). **p < 0.01 compared with negative or si-nc, ##*p* < 0.01 compared with ZDHHC16 or si-ZDHHC16.

Furthermore, we examined the mechanism of ZDHHC16 on the ferroptosis of DPSCs. In this study, we found that the ZDHHC16 plasmid triggered the expression of the ZDHHC16 protein and inhibited the expression of the p-CREB protein of the DPSCs **(**Fig. 4A**)**. Moreover, the Si-ZDHHC16 plasmid was found to exert a suppressive effect on ZDHHC16 protein expression while simultaneously inducing the expression of p-CREB protein in DPSCs **(**Fig. 4B**)**. Immunofluorescence showed that ZDHHC16 plasmid induced ZDHHC16 expression and suppressed p-CREB expression of DPSCs **(**Fig. 4C**)**. IP also showed that the ZDHHC16 protein interlinked with the CREB protein **(**Fig. 4D**)**. CREB agonist (10 µM of compound AE-18) induced p-CREB and GPX4 protein expressions of DPSCs by ZDHHC16 up-regulation **(**Fig. 4E**)**. The CREB inhibitor (100 µM of CREB-IN-1 TFA) suppressed p-CREB and GPX4 proteins of DPSCs by down-regulation of ZDHHC16 **(**Fig. 4F**)**. We confirmed that ZDHHC16 suppressed the p-CREB expression of DPSC.

### CREB regulated the ZDHHC16 effects on cell development of DPSCs

**Fig. 5:**
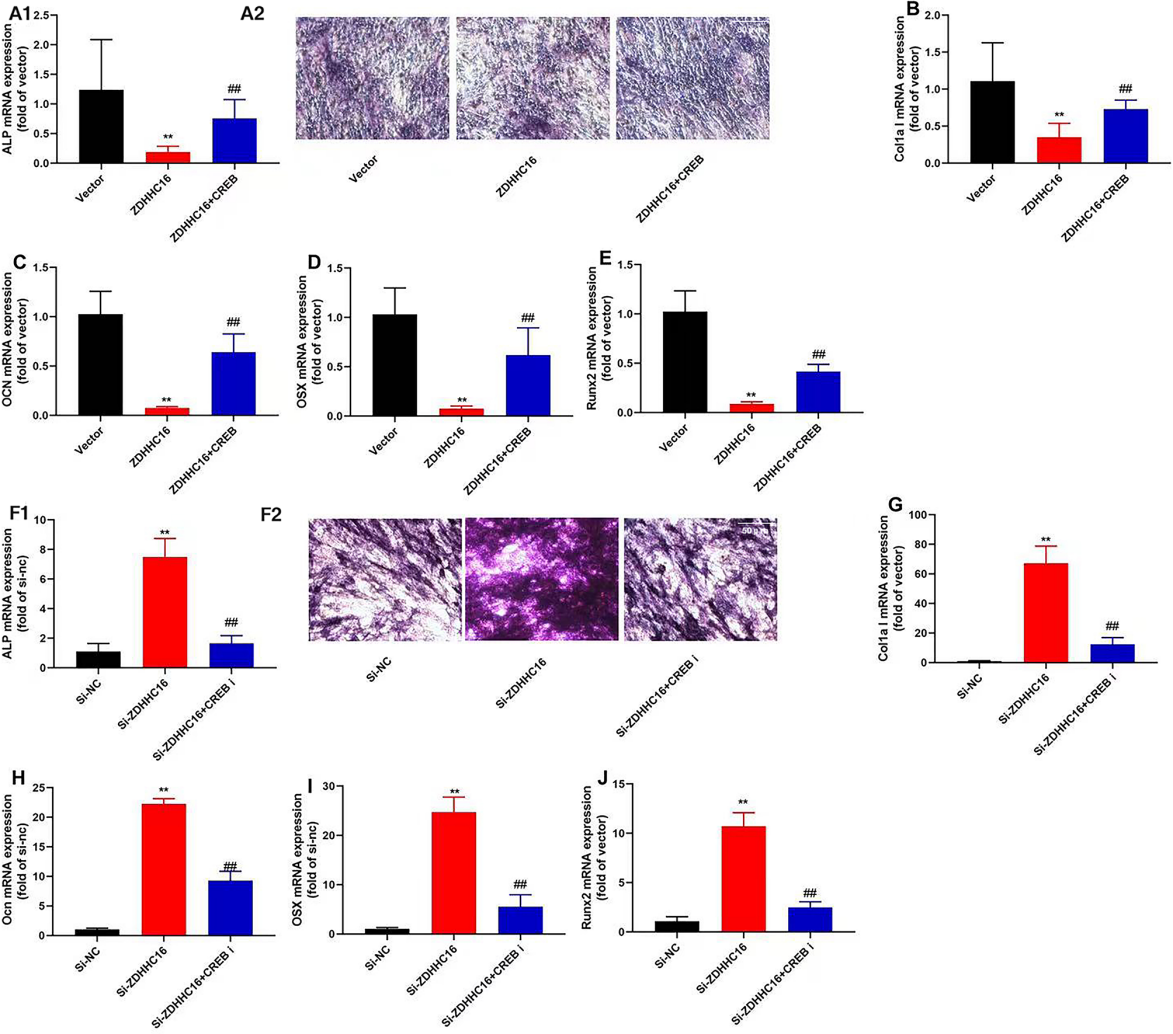
CREB regulated the effects of ZDHHC16 on cell growth of DPSCs CREB agonist promoted the cell growth (**A**), increased the total number of EDU cells (×20, B1 and B2), and enhanced the migration rate(×50, C1 and C2) of DPSCs by up-regulation of ZDHHC16 ; CREB suppressor reduced the cell growth (**D**), decreased the total number of EDU cells (×20, E1 and E2), and decreased the migration rate(×50, F1 and F2) of DPSCs by down-regulation of ZDHHC16. **p < 0.01 compared with negative or si-nc, ##*p* < 0.01 compared with ZDHHC16 or si-ZDHHC16.

Subsequently, an evaluation is conducted to determine the role of CREB in the impact of ZDHHC16 on DPSC cell proliferation. In the current study, we found that the administration of a CREB agonist, specifically 10 µM of compound AE-18, resulted in the promotion of cell growth and an increase in the total number of EDU cells. Additionally, the migration rate of DPSCs was enhanced through the upregulation of ZDHHC16 **(**Fig. 5A–C**)**. The application of a CREB suppressor (specifically, 100 µM of CREB-IN-1 TFA) resulted in a reduction in cell growth and a decrease in the total quantity of EDU cells. Additionally, the migration rate of DPSCs was observed to decrease due to the down-regulation of ZDHHC16 **(**Fig. 5D–F**)**.

### CREB regulated the effects of ZDHHC16 on the OD of DPSCs

**Fig. 6:**
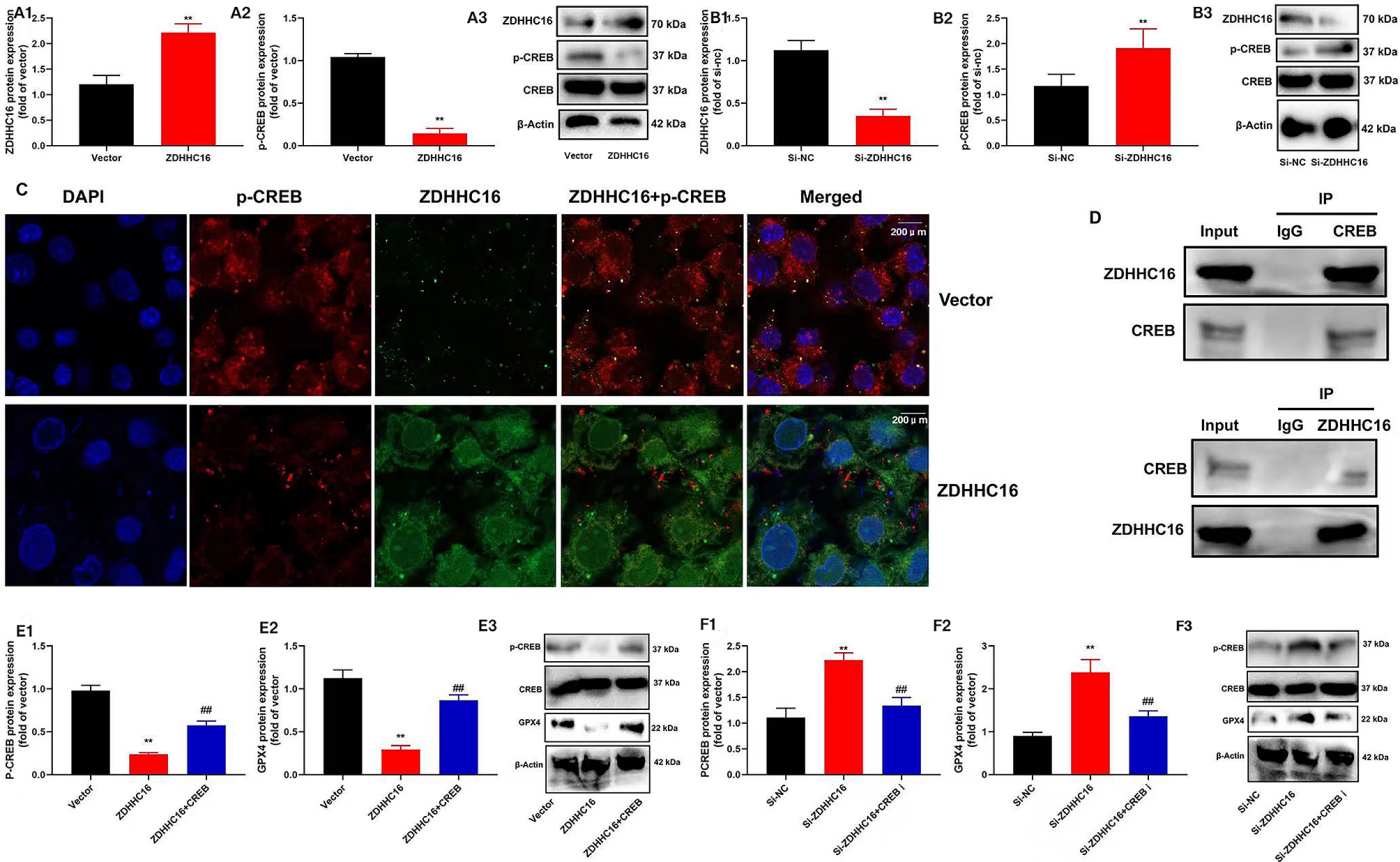
CREB regulated the effects of ZDHHC16 on Osteogenic differentiation of DPSCs CREB agonist promoted OD of DPSCs by ZDHHC16 up-regulation: ALP mRNA expression and staining (×50, A1,A2), Col1a1 (**B**), OCN (C), OSX (**D**) and Runx2 mRNA expression(**E**); CREB inhibitor reduced OD of DPSCs by down-regulation of ZDHHC16: ALP mRNA expression and staining (×50, F1, F2), Col1a1 (**G**), OCN (**H**), OSX (**I**) and Runx2 (**J**) in DPSCs by ZDHHC16 down-regulation. **p < 0.01 compared with negative or si-nc, ##*p* < 0.01 compared with ZDHHC16 or si-ZDHHC16.

Consequently, we assessed the function of CREB in the effects of ZDHHC16 on the osteogenic differentiation of DPSCs. CREB agonist (10 µM of compound AE-18) promoted OD of DPSCs by ZDHHC16 up-regulation **(**Fig. 6A–E**)**. The CREB inhibitor (100 µM of CREB-IN-1 TFA) reduced osteogenic differentiation of DPSCs by down-regulation of ZDHHC16 **(**Fig. 6F–J**)**.

### CREB inhibitor reversed the effects of ZDHHC16 on ferroptosis of DPSCs

**Fig. 7:**
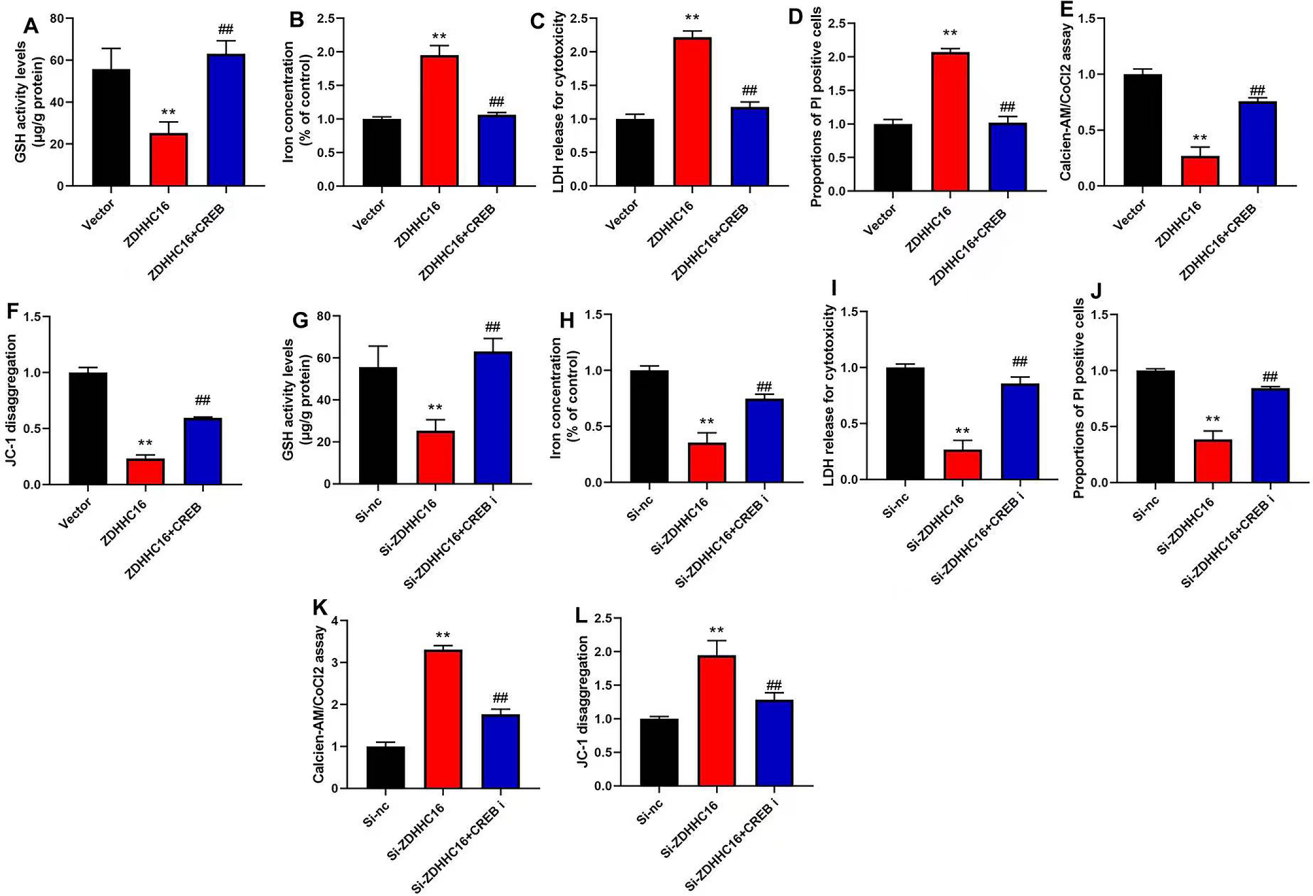
CREB regulated the effects of ZDHHC16 on Ferroptosis of DPSCs CREB agonist promoted GSH activity levels (**A**), reduced iron concentration (**B**) and LDH activity levels (**C**), inhibited PI-positive cells (**D**), increased MPT (**E**) and JC-1 disaggregation (**F**) of DPSCs by ZDHHC16 up-regulation; CREB inhibitor reduced GSH activity levels (**G**), increased iron concentration (**H**) and LDH activity level (**I**), promoted PI-positive cells (**J**), and suppressed MPT (**K**) and JC-1 disaggregation (**L**) of DPSC by ZDHHC16 down-regulation. **p < 0.01 compared with negative or si-nc, ##*p* < 0.01 compared with ZDHHC16 or si-ZDHHC16.

Lastly, we further assess the role of CREB in the effects of ZDHHC16 on the ferroptosis of DPSCs. We found that CREB agonist (10 µM of compound AE-18) promoted GSH activity levels, reduced iron concentration and LDH activity levels, inhibited PI-positive cells, increased JC-1 disaggregation and MPT (calcein AM/CoCl2 assay) of DPSCs by ZDHHC16 up-regulation **(**Fig. 7A–F**)**. Additionally, CREB inhibitor (100 µM of CREB-IN-1 TFA) reduced GSH activity levels, increased iron concentration and LDH activity level, promoted PI-positive cells, and suppressed JC-1 disaggregation and MPT (calcein AM/CoCl2 assay) of DPSC by ZDHHC16 down-regulation **(**Fig. 7G–L**)**.

## Discussion

Periodontitis is an inflammatory disease caused by the interaction between plaque microorganisms and the host autoimmune system. Alveolar bone resorption caused by periodontitis is the leading cause of tooth loss in adults. The key to the treatment of periodontitis is to inhibit bacterial inflammation on the one hand and promote the regeneration of periodontal tissue on the other hand [33]. Several techniques for reconstructing bones have been employed in the restoration of maxillofacial abnormalities. Recently, an increasing number of investigations have indicated that bone tissue engineering presents a viable substitute for conventional autologous, allogeneic, and xenogeneic bone grafts [34].

The use of stem cells for tissue engineering in alveolar bone regeneration is a promising method. For bone regeneration, DPSCs were most studied [35]. Additionally, multiple research studies have demonstrated the effectiveness of utilizing DPSCs in conjunction with biomaterials as a successful approach for reconstructing bone defects and promoting craniofacial bone regeneration [36]. The specific regulatory mechanisms governing the OD of DPSCs are not yet fully understood, however. A recent study showed that palmitic acid was toxic to osteoblasts in cell culture models [37]. We observed that the increased expression of ZDHHC16 resulted in reduced cell development (Fig. 1B), a decrease in the number of EdU-labeled cells (Fig. 1C1, C2), and an inhibition of the migration rate of DPSCs (Fig. 1D1, D2). Conversely, the down-regulation of ZDHHC16 enhanced the migration rate of DPSCs (Fig. 1H1, H2) and led to increased cell growth (Fig. 1F) and a higher quantity of EdU-labeled cells (Fig. 1G1, G2). Herein, we found that inhibition of ZDHHC16 promoted DPSCs cell growth. The preservation of tissue and organ homeostasis, as well as regeneration, depends on the intricate coordination of cellular processes, encompassing proliferation, migration, adhesion, and differentiation [38]. Therefore, ZDHHC16 could be crucial for the cell growth of DPSCs.

DPSCs exhibit comparable immunophenotypic and pluripotent differentiation features to BMSCs [39]. It is informed that the osteogenic capacity of inflammatory DPSCs is lower than that of normal DPSCs, but understanding its mechanism is still limited. Clarifying the molecular pathway of inhibition of OD of DPSCs in an inflammatory environment is crucial for finding effective drugs for tooth regeneration [40]. Protein S-palmitoylation, a conserved modification observed in all eukaryotic cells, plays a crucial role in regulating protein stability, enzymatic activity, protein trafficking, and various other cellular processes during post-translational processing. Therefore, a multitude of human diseases are associated with a disruption in the equilibrium of protein S-palmitoylation, particularly in the context of malignancies and neurological disorders [41]. It remains unclear how palmitoylation contributes to osteogenesis. Researchers have found that ZDHHC13 regulates bone homeostasis as a palmitoyl acetyltransferase [42]. In this mouse model, ZDHHC13 promoted the acquisition of bone mass and contributes to postnatal skeletal development. Palmitic treatment suppressed the expression of ALP, OCN, and Runx2, consequently hindering OD. Additionally, palmitic treatment significantly reduced the levels of Zdhhc1, Zdhhc2, and Zdhhc12, indicating that palmitoylation played an inhibitory role in the osteogenesis of osteoblasts. In our study, we found that palmitoylation mediated by ZDHHC16 inhibited OD using a DPSCs culture model (Fig. 2A–J). The aforementioned findings corroborate our hypothesis that ZDHHC16 plays a crucial role in the OD process of DPSCs. In vitro studies have demonstrated that the incorporation of cytokines facilitates the conversion of DPSCs from multipotent stem cells to osteoblasts [43]. Some nerve growth factors and particular proteins generated during osteogenesis have been discovered to augment OD of DPSCs further by utilizing shared signaling pathways [44]. So S-palmitoylation may also be a regulatory factor in OD of DPSCs. Whether ZDHHC16 has the same impact on the OD of DPSCs in an inflammatory environment requires further study.

Recent research has redefined the periodontitis classification, showing a crucial role for regulated cell death in the disease [45]. The core characteristics of ferroptosis are the formation of high-level iron-catalyzed free radicals in cells, the accumulation of unsaturated fatty acids, and the accumulation of iron-induced lipid active oxygen species, which cause oxidative stress and damage to DNA, protein, and lipid [13]. The level of iron in periodontitis patients increases. At the same time, there are abnormalities in the intracellular antioxidant system, mainly manifested by a decrease in GPX4 activity. Although iron overload is considered a risk factor for periodontal disease, it is not yet known whether the iron concentration in patients with periodontal disease can lead to the occurrence of related pathological processes [46]. Qiao et al. suggesting that the inhibition of ferroptosis could serve as a novel therapeutic approach for managing the occurrence and progression of periodontitis [47]. Reducing the degree of ferroptosis in DPSCs, restoring cellular homeostasis, is also beneficial for their OD and anti-inflammatory activity [48]. The enhancement of cellular free iron levels and the promotion of DPSCs proliferation in hypoxia are facilitated by nuclear receptor coactivator 4 (NCOA4)-mediated ferritinophag [16]. We identified that inhibition of ZDHHC16 reduced DPSCs ferroptosis (Fig. 3A–H), demonstrating that it may be a treatment target for DPSCs OD or periodontitis treatment by regulation of ferroptosis.

CREB is an important transcription factor that depends on phosphorylation and plays a vital function in cell growth, survival, and tumor metastasis. Inhibition of CREB phosphorylation is an important way to suppress the overgrowth of chronic myeloid leukemia cells and promote their apoptosis. The hypothesis of neural plasticity involved in the CREB signal pathway has received more and more attention. CREB enhances protein kinase A (PKA) activity in specific parts of the brain [49]. PKA is widely distributed in stem cells to activate the transcription reaction of the downstream signal pathway [50]. CREB is a phosphorylated transcription factor; as an intersection point of the intracellular signal pathway related to periodontitis, it can regulate multiple stem cell functions and further regulate the activity of brain derived neurotrophic factor to change the response of external cells to stimulation. Also, CREB inhibited ferroptosis in lung adenocarcinoma [51]. The activated CREB binds to the cAMP response element (CRE) and triggers osteogenesis-related gene expression. However, other post-translational modifications of CREB are rare [52]. In this study, we found that ZDHHC16 protein exhibited interconnection with CREB, and ZDHHC16 suppressed p-CREB expression (Fig. 4A-F). We hypothesize that the interaction between CREB and ZDHHC16 disrupts (PKA) binding to CREB, resulting in diminished phosphorylation. The investigation into the underlying mechanism warrants further exploration.

The OD of DPSCs is an intricate process governed by the modulation of several signaling pathways, such as the Wnt/β-catenin signaling pathway and the mitogen-activated protein kinase (MAPK) pathway. The CREB pathway, through its involvement in Wnt5a, is capable of upregulating Runx2 and downregulating peroxisome proliferator-activated receptor γ(PPARγ) expression. This effectively enhances OD of DPSCs [53] while inhibiting adipogenic differentiation [54]. Therefore, our study also supports this viewpoint, namely, ZDHHC16 suppressed the expression of p-CREB to reduce OD of DPSCs and DPSCs ferroptosis by CREB (Figs. 5, 6 and 7). So ZDHHC16 may be a marker of OD and a potential treatment target for osteogenic differentiation of DPSCs or the treatment of alveolar bone defects by periodontitis. There is a need for further research into the regulatory mechanism between upstream and downstream signaling pathways. Subsequent investigations will be conducted by our research team to examine the potential of ZDHHC16-mediated regulation of exosomes derived from mesenchymal stem cells to augment the optical density of DPSCs.

This study is the first experiment to reveal the relationship between ZDHHC16 and OD of DPSCs. But it has some limitations. First of all, we investigated the relationship between ZDHHC16 and OD of DPSCs, while other DHHC-containing palmitoyl transferases may also be involved in this process. Besides, we only studied DPSCs under physiological conditions, and the results of DPSCs under infection conditions may be different.

## Conclusions

Our research conducted the initial experimental study about the relationship between ZDHHC16 and DPSCs, indicating that the suppression of ZDHHC16 facilitated the OD of DPSCs by inhibiting ferroptosis through CREB.

### Electronic supplementary material

Below is the link to the electronic supplementary material.

Supplementary Material 1

## Data Availability

The datasets generated and analyzed during the current study are not publicly available due to privacy restrictions but are available from the corresponding author on reasonable request.

## Abbreviations

BMSCs: Bone marrow mesenchymal stem cells
CLDN3: claudin 3
Col1a1: Collagen type I alpha 1 chain
CREB: cAMP-response element binding protein
DHHC: Asp-His-His-Cys
DPSCs: Dental pulp stem cells
DMEM: Dulbecco’s Modified Eagle Medium
EdU: Ethynyl deoxyuridine staining
FBS: fetal bovine serum
OD: Osteogenic differentiation
OCN: Osteocalcin
OSX: Osterix
GPX4: Glutathione peroxidase 4
GSH: Glutathione
MAPK: mitogen-activated protein kinase
NCOA4: Nuclear receptor coactivator 4
IP: Immunoprecipitation
PPARγ: Peroxisome proliferator-activated receptorγ
ROS: Reactive oxygen species
Runx2: Runt-related transcription factor 2
SETD2: SET domain-containing 2
SLC7A11: Solute carrier family 7 member 11
ZDHHC16: Zinc finger DHHC-type palmitoyltransferases
