# Supplementary material for: Inhibition of ZDHHC16 promoted osteogenic differentiation and reduced ferroptosis of dental pulp stem cells by CREB
Source: BMC Oral Health. 2024 Mar 26;24:388. doi: 10.1186/s12903-024-04107-x (PMC10964552; doi:10.1186/s12903-024-04107-x)
Supplement: Supplementary file 1 — Supplementary Material 1 [file 12903_2024_4107_MOESM1_ESM.pdf]

β-Actin 42 kDa

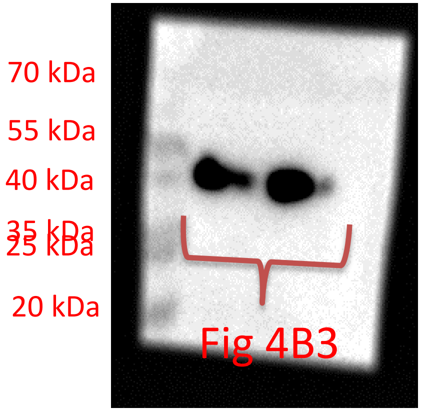

β-Actin 42 kDa

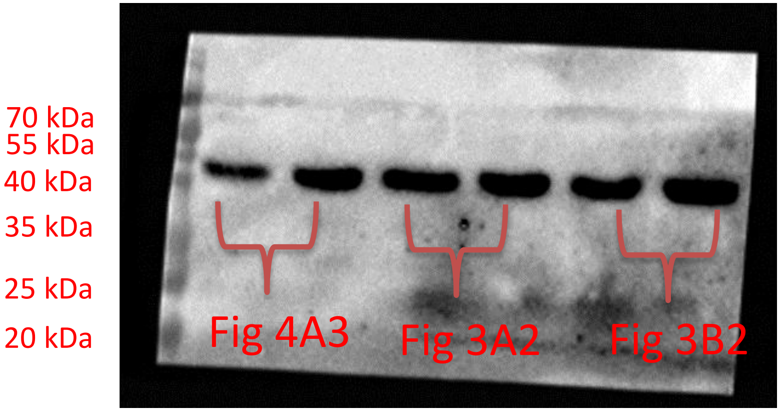

β-Actin 42 kDa

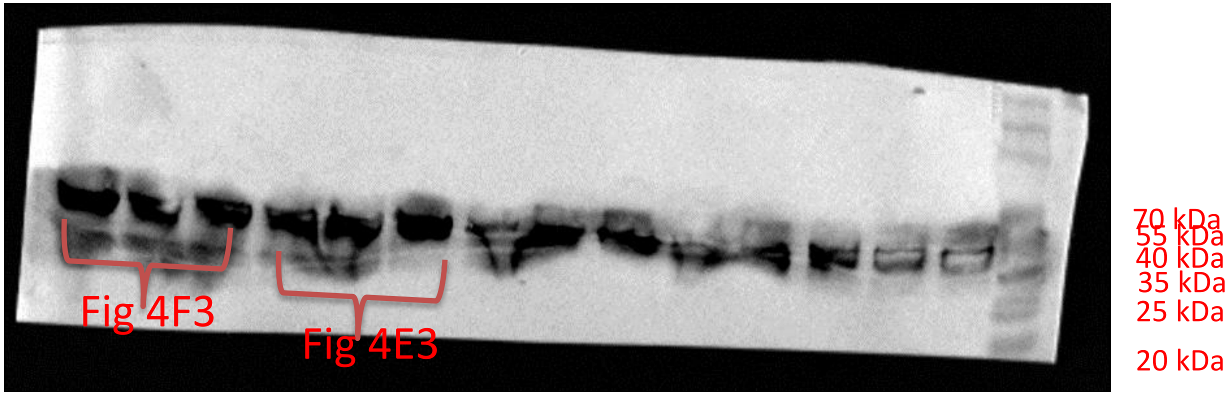

ZDHHC16 70 kDa

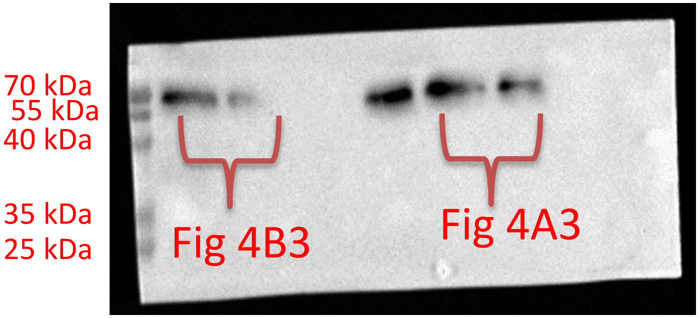

IP: ZDHHC16 70 kDa Fig 4D

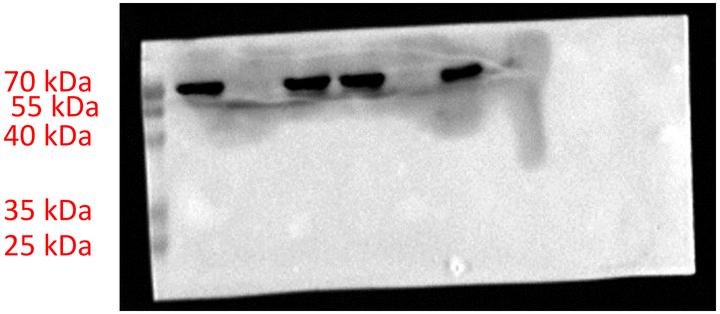

GPX4 22 kDa

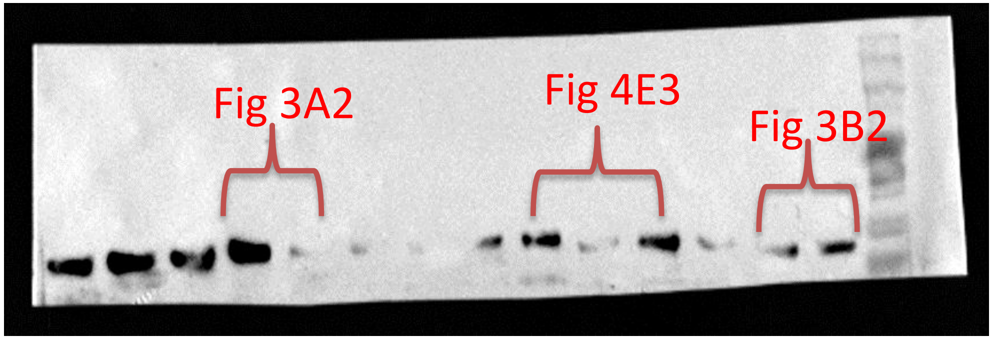

GPX4 22 kDa

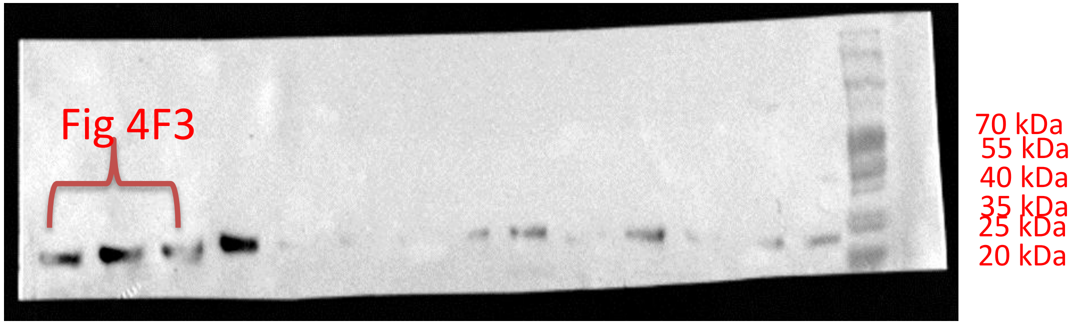

CREB 37 kDa

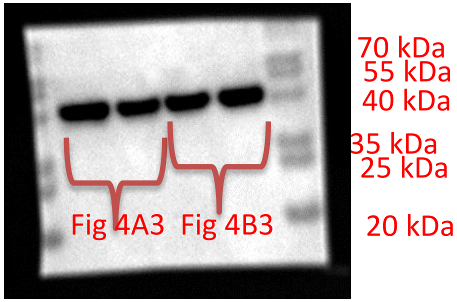

CREB 37 kDa

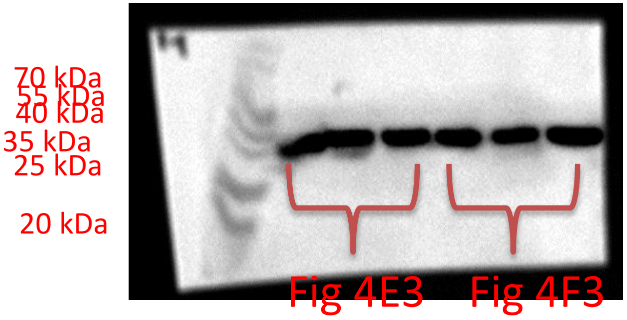

IP: CREB 37 kDa Fig 4D

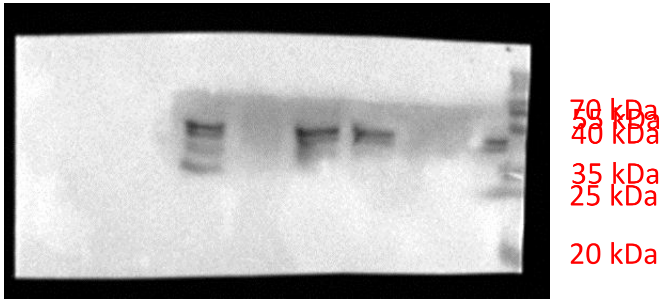

pCREB 37 kDa

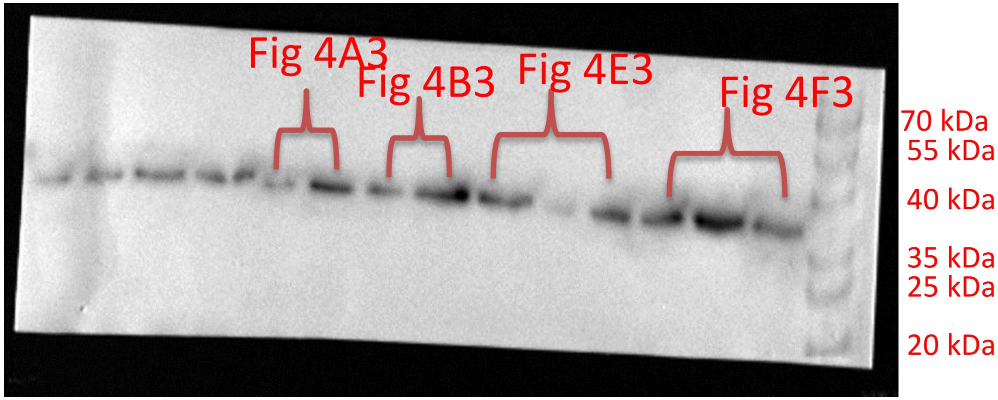

Fig 4A3
Fig 4B3
Fig 4E3
Fig 4F3
70 kDa
55 kDa
40 kDa
35 kDa
25 kDa
20 kDa
